# Photobiomodulation enhanced endogenous pain modulation in healthy volunteers

**DOI:** 10.1007/s10103-022-03686-x

**Published:** 2022-12-23

**Authors:** Yuka Oono, Ryoko Kono, Yuki Kiyohara, Saori Takagi, Yasuo Ide, Hiroshi Nagasaka, Hikaru Kohase

**Affiliations:** 1 Division of Dental Anesthesiology, Department of Diagnostic and Therapeutic Sciences, Meikai University School of Dentistry, Saitama, Japan; 2 Department of Anesthesiology, Saitama Medical University Hospital, Saitama, Japan

**Keywords:** Photobiomodulation, Acupuncture therapy, Diffuse noxious inhibitory control, Pain management

## Abstract

To examine the effects of photobiomodulation (PBM) in healthy volunteers using photonic stimulation of acupuncture points on conditioned pain modulation (CPM), temporal summation of pain (TSP), and offset analgesia (OA), which reflect some aspects of endogenous pain modulation. We included 15 men and 15 women (age, 31.5 [27.3–37.0], body mass index, 25.7 [24.4–27.1], Fitzpatrick skin typing, II: 20, III: 8, IV: 2). CPM, TSP, and OA were evaluated after a sham procedure (control session) and after acupuncture point stimulation (LI4 and LI10 on the non-dominant forearm) using linear polarized near-infrared light irradiation (LPNILI; wavelengths peaked at approximately 1000 nm, output: 1.4 W/cm^2^, spot diameter: 10 mm, spot size: 1.02 cm^2^, maximum temperature: 40.5 °C, pulse width: 1 s, frequency: 0.2 Hz) (PBM session). Differences in CPM, TSP, and OA between the two sessions were evaluated by the paired *t*-test and Fisher’s exact test (statistical significance: *p* < 0.05). Values indicate median [interquartile range]. LPNILI significantly increased CPM in all participants (control session: 12.1 [−4.5–37.4], PBM session: 23.9 [8.3–44.8], *p* < 0.05) and women (control session: 16.7 [−3.4–36.6], PBM session: 38.7 [24.6–52.1], *p* < 0.05). The CPM effect increment was significantly higher in women than in men (*p* = 0.0253). LPNILI decreased TSP in participants with higher TSP ratios (*p* = 0.0219) and increased OA in participants with lower OA scores (*p* = 0.0021). LPNILI enhanced endogenous pain modulation in healthy volunteers, particularly in women, as evaluated using CPM. CPM, TSP, and OA evaluations are potentially useful for discriminating PBM responders from non-responders.

## Introduction

Photobiomodulation (PBM) is useful for pain management [1]. In such instances, a phototherapeutic device with linear polarized near-infrared light (LPNIL) is used for PBM [2]. LPNIL is generated by a linear polarizer, and it generates an electric field perpendicular to oscillation [2]. An LPNIL irradiator emits rays with a wavelength of 600–1600 nm which can deeply penetrate the subcutaneous tissue [2]. PBM is effective for pain relief because it inhibits the action potentials in the dorsal roots [3] and nociceptive signals at peripheral nerves [4]. PBM promotes wound healing, reduces pain, and accelerates the anti-inflammatory process [1–4]. In addition, PBM is used to treat refractory orofacial pain, such as trigeminal neuralgia [5]. However, the response to PBM differs between individuals [1, 5, 6]; some individuals respond well to PBM therapy, while others do not show an adequate response. The absorption process of red/near-infrared light energy, which enhances mitochondrial adenosine triphosphate production, cell signaling, growth factor synthesis, and attenuates oxidative stress, is termed “PBM” [6].

Acupuncture is an effective treatment for chronic pain and has long-term effects [7]; it might also positively affect primary trigeminal neuralgia [8]. The acupuncture points for trigeminal neuralgia or facial nerve palsy include Hegu (LI4), LI20, ST2, ST4, and ST7 [9]. A systematic review indicated that laser acupuncture therapy (LAT) improves pain and functional outcomes [10]. Laser acupuncture is defined as “photonic stimulation of acupuncture points and areas to initiate therapeutic effects similar to that of needle acupuncture and related therapies together with the benefits of PBM” [11].

Conditioned pain modulation (CPM) is a phenomenon through which the conditioning stimulus affects the test stimulus [12]. CPM is a centrally processed measure of the net effect of the descending pain pathway [12], including serotonergic and noradrenergic neurons [13–15]. When exploring CPM, a test stimulus (e.g., pressure pain thresholds [PPTs]) and a conditioning stimulus (e.g., thermal stimulus) are applied [13, 16]. The change in the perceived test stimulus induced by the conditioning stimulus is reported as the CPM effect [12].

Temporal summation of pain (TSP) is a process of increased pain sensation triggered by repetitive stimulation such as a pinprick or thermal stimulation [17]. TSP is calculated by the subject’s pain rating of the repetitive stimuli. It is observed in healthy humans and is enhanced in pain patients compared to healthy humans [17]. TSP is considered to reflect central sensitization, attenuated by opioid agonists and N-methyl-D-aspartate receptor antagonists [18–20].

Offset analgesia (OA) is a large reduction in pain after a brief increase in the intensity of a painful thermal stimulus [21]. The OA condition includes temperature deviations (+1 °C; e.g., 48 °C) from an initial temperature (e.g., 47 °C), resulting in a hypoalgesic response. OA is usually evaluated as a change in the visual analogue scale (VAS) values for a thermal stimulus. OA is associated with reward systems and descending pain modulatory systems [21].

CPM, TSP, and OA are commonly used paradigms that reflect some aspects of endogenous pain modulation [22], with different underlying physiological mechanisms [23]. We previously presented a case report in which the triggered pain of trigeminal neuralgia was attenuated by irradiation of acupuncture points (LI4, LI20, ST2, and ST7, in addition to trigger points) with a semiconductor laser by facilitating descending inhibitory controls [24]. The CPM effect increased after laser irradiation [24]. A previous case report of a patient with trigeminal neuralgia suggested that one possible mechanism for pain reduction is the facilitation of descending inhibitory controls via photonic stimulation of acupuncture points [24].

CPM, TSP, and OA evaluations are potentially useful for discriminating responders from non-responders to PBM and LAT. However, no previous study has assessed endogenous pain modulation using CPM, TSP, and OA with photonic stimulation of acupuncture points. Therefore, the aim of this study was to investigate the effects of PBM via photonic stimulation of acupuncture points on CPM, TSP, and OA in healthy volunteers.

## Materials and methods

### Participants

This study was conducted at the Division of Dental Anesthesiology, Department of Diagnostic and Therapeutic Sciences, Meikai University School of Dentistry. Written informed consent was obtained from all participants before they were included in the study. This study was conducted in accordance with the Declaration of Helsinki and approved by the Ethics Committee of Meikai University (A1934). Moreover, the study was registered with the University Hospital Medical Information Network Clinical Trials Registry (UMIN-CTR) as a clinical trial (unique ID: UMIN000039812) on March 13, 2020.

The inclusion criteria were as follows: (1) >18 years old; (2) healthy and pain-free individuals; and (3) individuals who could provide informed consent. The exclusion criteria were as follows: (1) the presence of a serious medical condition, such as acute or chronic pain and neurological, psychiatric, or neuromuscular diseases; (2) current use of any pain medication within 24 h prior to the investigation; and (3) the inability to provide informed consent.

### CPM evaluation

PPTs were evaluated in the dominant forearm with and without a thermal conditioning stimulus as a test stimulus to assess CPM potency [16, 24–27]. PPT was assessed using a customized electronic pressure algometer (AIKOH Engineering, Osaka, Japan) with a probe area of 1 cm^2^ [24, 25, 27]. PPT is the amount of pressure (N) perceived as painful by the participant. The pressure was applied at a steadily increasing rate of 3 N/s (30 kPa/s) [16, 24–27]. The participant pressed the stop button when the threshold was reached. PPT measurements were repeated thrice at 1-min intervals. The mean value of three recordings was used for subsequent analysis. PPTs were recorded before (baseline) and during the conditioning stimulus. The conditioning stimulus was tonic painful cold–heat pulse stimulation consisting of a sequence of repeated alternating cold (−10 °C) and heat stimulations (47 °C) delivered at 20-s intervals (0.025 Hz) using a customized quantitative thermal stimulator device (VTH-3500, VICS, Tokyo, Japan) [25, 27, 28]. A tonic painful cold–heat pulse stimulus was applied to the non-dominant forearm [25, 27]. The application of the conditioning stimulus began 2 min before the test stimulus until the end of the PPT measurement period (for 5 min). To ensure a constant attention level of the participant throughout the experiment, they were instructed to focus on their PPT. The CPM effect was calculated as follows: (PPT with conditioning stimulus/PPT without conditioning stimulus − 1) × 100 (%) [27]. Additionally, the increase in the CPM effect by the photonic stimulation of acupuncture points was defined as follows: (CPM effect in PBM session) − (CPM effect in control session).

### TSP evaluation

The thermal stimulation was applied by a customized quantitative thermal stimulator device (VTH-3500, VICS, Tokyo, Japan) to induce TSP [25, 27, 28]. Thermal stimulation of 47 °C (2 s) was repeated 10 times (0.3 Hz) to the dominant forearm. For the continuous evaluation of subjective assessments of pain intensity induced by thermal stimulation, the participants were asked to continuously rate the pain intensity for thermal stimulation using a custom-made electronic VAS (0–100 mm). The custom-made electronic VAS, employing a sliding resistor, was applied, and VAS values were sampled and analyzed by a personal computer [25, 27, 28]. The left endpoint (0) of the electronic VAS indicated “no pain,” and the right endpoint (100) indicated the “worst pain imaginable.” The TSP ratio was calculated as follows: mean VAS scores from the 8th to the 10th stimulus/mean VAS scores from the first to the fourth stimulus [27].

### OA evaluation

OA was evaluated at the dominant forearm and evoked by a three-heat-stimulus train (T1–T2–T3), with T1, T3 (46 °C), and T2 (47 °C), which were applied by a customized quantitative thermal stimulator device (VTH-3500, VICS, Tokyo, Japan) [25, 27, 28]. The baseline temperature was 32 °C, and T1 (46 °C) and T2 (47 °C) lasted for 5 s, while T3 (46 °C) lasted for 20 s. The participants were asked to continuously rate the pain intensity for thermal stimulation using a custom-made electronic VAS (0–100 mm), which was sampled and analyzed using a personal computer [25, 27, 28]. The OA score was defined as follows: (maximum VAS score for T2) – (minimum VAS score for T3) [27].

### Device for CPM, TSP, and OA evaluation

For the thermal stimulation in CPM, TSP, and OA evaluation, a customized quantitative thermal stimulator device (VTH-3500, VICS, Tokyo, Japan) was applied to the non-dominant forearm in CPM and the dominant forearm in TSP and OA [25, 27, 28]. The quantitative thermal stimulator device consisted of a ceramic contact plate (30 × 30 mm) that was cooled or heated with a Peltier element. The temperature was continuously measured using a thermometer placed on the surface of the Peltier element [25, 27, 28].

### Photonic stimulation

An output spectrum from LPNIL (Super Lizer mini PRO^TM^, Tokyo Iken Co., Ltd, Tokyo, Japan) was applied at wavelengths ranging from 600 to 1600 nm; the strongest wavelength was approximately 1000 nm [2]. The Super Lizer mini PRO^TM^ is a spot-irradiation-type LPNIL irradiator that uses a halogen lamp; the irradiation unit can stimulate one point on the skin, and the maximum output power is 2.4 W/cm^2^. The participants were exposed to infrared light (output: 1.4 W/cm^2^ [which is 60% of 2.4 W/cm^2^, the maximum output power], spot diameter: 10 mm, spot size: 1.02 cm^2^, maximum temperature: 40.5 °C, pulse width: 1 s, and frequency: 0.2 Hz [with an on–off time of 1–4 s]). The linear polarized near-infrared light irradiation (LPNILI) was applied to the non-dominant forearm, and the photonic acupuncture points were LI4 and Shousanli (LI10). The distance between the tip of the irradiation probe and the skin surface was measured and set at 1 mm and maintained by the probe holder. The duration for irradiation was 10 min for LI4 and 10 min for LI10 (a total of 20 min). LI4 and LI10 were not stimulated simultaneously because the device was equipped with one probe. The order of the irradiation for LI4 and LI10 was randomized. Before irradiation of LI4 and LI10, the device was set to the above-mentioned conditions (output: 1.4 W/cm^2^, spot diameter: 10 mm, maximum temperature: 40.5 °C, pulse width: 1 s, frequency: 0.2 Hz [an on–off time of 1–4 s], and duration: 10 min). The device does not require calibration.

In the control session, the probe was set 1 mm from the skin surface using the probe holder as the PBM session. The sham procedure was performed at LI4 for 10 min and LI10 for 10 min without irradiation.

### Protocol

All experiments were performed at a constant room temperature (25 °C). All participants underwent two experimental sessions: a control session followed by a 1-h break and then a PBM session (both sessions occurred on the same day).

In the control session, CPM, TSP, and OA were evaluated at the dominant forearm after the sham procedure (without LPNILI of acupuncture points on the non-dominant forearm). The sham procedure without irradiation was performed at LI4 for 10 min and LI10 for 10 min (the order of LI4 and LI10 was randomly assigned) with a phototherapeutic device with LPNIL (Super Lizer mini PRO^TM^, Tokyo Iken Co., Ltd, Tokyo, Japan). The order of CPM, TSP, and OA evaluations was randomly assigned.

In the PBM session, CPM, TSP, and OA were evaluated at the dominant forearm after LPNILI of acupuncture points on the non-dominant forearm (LI4 for 10 min and LI10 for 10 min, the irradiation order was randomly assigned) with a phototherapeutic device with LPNIL (Super Lizer mini PRO^TM^, Tokyo Iken Co., Ltd, Tokyo, Japan). The order of CPM, TSP, and OA evaluations was randomly assigned.

YK carried out the sham and light irradiation. RK carried out the CPM, TSP, and OA evaluation. YO carried out the data analysis. Participants and examiners for CPM, TSP, and OA assessment (RK) were blinded for the sham irradiation (control session) and irradiation (PBM session).

The irradiated parameters (output, duration, pulse width, and frequency) were created and set by the device, and the distance between the tip of the irradiation probe and the skin surface was measured and maintained by the probe holder. These settings were implemented before irradiation by YK.

### Statistical analyses

The F-test for homogeneity of variance and the one-sample Kolmogorov–Smirnov test were performed before the unpaired and paired *t*-tests. The unpaired *t*-test was used to analyze gender differences in participants’ age and body mass index (BMI). The paired *t*-test was conducted to analyze differences in the CPM effect, TSP ratio, and OA score between control and PBM sessions. Additionally, the changes in CPM, TSP, and OA by irradiation were evaluated by comparing the results from control and PBM sessions. The relationship between the photonic stimulation effect of acupuncture points and the CPM effect, TSP ratio, and OA score was analyzed using Fisher’s exact test. Statistical significance was set at *p* < 0.05. Statistical analyses were performed using EZR (version 1.54) [29]. Values (age, weight, height, BMI, CPM effect, TSP ratio, and OA score) are presented as median [interquartile range].

### Sample size calculation

An *a priori* power analysis was performed to establish the necessary sample size for this study using G*Power (version 3.1.9.7) [30] with a probability of type I error of 0.05, a power of 0.8, and an effect size of 0.5. Based on these parameters, the power analysis demonstrated that a total sample size of 27 was required for this study. Since some participants may drop out due to various reasons, the initial sample size was increased by 10%. The final sample size was 30 for this study.

## Results

A total of 15 men and 15 women (median age 31.5 years [27.3–37.0]) participated in this study (Table 1). There were no significant differences (*p* > 0.05) in the homogeneity of variance and normality for age, BMI, the CPM effect, TSP ratio, or OA score. Additionally, the unpaired *t*-test showed no significant gender differences in age and BMI (*p* > 0.05).

**Table 1:** Demographic data of the participants

	Men	Women	All
Number of participants	15	15	30
Age (years)	30.0 [27.5–32.5]	36.0 [27.0–40.5]	31.5 [27.3–37.0]
Weight (kg)	64.0 [61.0–71.0]	53.0 [49.0–55.0]	55.0 [53.0–64.0]
Height (cm)	173.0 [168.5–176.5]	158.0 [157.0–158.0]	161.0 [158.0–173.0]
BMI	25.8 [24.8–27.5]	25.5 [23.8–26.4]	25.7 [24.4–27.1]
Fitzpatrick skin typing	II: 9, III: 4, IV: 2	II: 11, III: 4	II: 20, III: 8, IV: 2

Median [interquartile range]

*BMI*, body mass index

### CPM effect

**Fig. 1:**
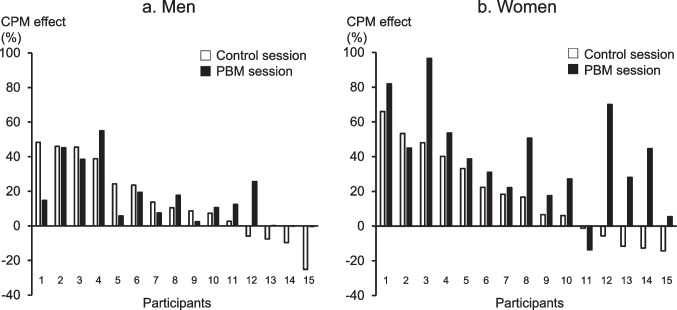
Frequency plots of individual CPM effects in the control session and PBM session in men (**a**) and women (**b**). Participants are plotted in the order of increasing CPM effects in the control session. White square: control session, solid square: PBM session; CPM, conditioned pain modulation; PBM, photobiomodulation

The CPM effects on all participants, men, and women are presented in Table 2. The paired *t*-test showed no significant difference between the CPM effects in the control and PBM sessions in men (*p* = 0.6068). However, a significant increase was observed in CPM effects in the PBM session compared with the control session in all participants (*p* = 0.0066) and women (*p* = 0.0035). The frequency plots of individual CPM effects in the control session and PBM session in men and women are shown in Fig. 1. The increase in the CPM effect by irradiation in men and women is presented in Table 3. This increase in the CPM effect was classified into a CPM effect ≥10% and a CPM effect <10%. Three of the 15 male participants had an increase in the CPM effect by over 10% by irradiation. In contrast, 10 of the 15 female participants had an increase in the CPM effect by over 10% by irradiation. Fisher’s exact test revealed that the magnitude of the CPM effect was increased by irradiation in women (*p* = 0.0253).

**Table 2:** CPM effect in the control session and PBM session

	Control session	PBM session
All (*n* = 30)	12.1 [−4.5–37.4]	23.9 [8.3–44.8]*
Men (*n* = 15)	10.5 [−1.6–31.6]	12.4 [4.1–22.5]
Women (*n* = 15)	16.7 [−3.4–36.6]	38.7 [24.6–52.1]*

Median [interquartile range]

^*^p < 0.05 compared with the control session

*CPM*, conditioned pain modulation; *PBM*, photobiomodulation

**Table 3:** Increase in the CPM effect by irradiation in men and women

	Increase in CPM effect ≥10 %	Increase in CPM effect <10 %
Men (*n* = 15)	3	12
Women (*n* = 15)	10	5

*p* = 0.0253 (Fisher’s exact test)

Increase in CPM effect by photonic stimulation of acupuncture points = (CPM effect in the PBM session) – (CPM effect in the control session)

*CPM*, conditioned pain modulation; *PBM*, photobiomodulation

### TSP ratio

**Fig. 2:**
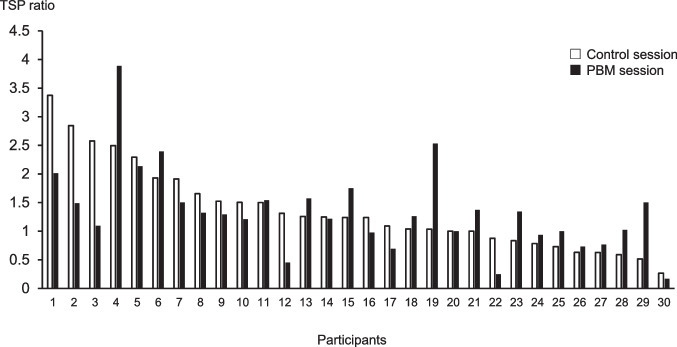
Frequency plot of individual TSP ratios for 30 participants. Participants are plotted in the order of increasing TSP ratios in the control session. White square: control session, solid square: PBM session; TSP, temporal summation of pain; PBM, photobiomodulation

The TSP ratios in all participants, men, and women are presented in Table 4. The paired *t*-test showed no significant difference between the TSP ratio in the control session and PBM session in all participants (*p* = 0.8961), men (*p* = 0.1794), and women (*p* = 0.0513). The frequency plots of individual TSP ratios in the control and PBM sessions in all participants are illustrated in Fig. 2. A decrease in the TSP ratio in the PBM session was observed in participants with high TSP ratios in the control session.

**Table 4:** TSP ratio in the control session and PBM session

	Control session	PBM session
All (*n* = 30)	1.2 [0.8–1.6]	1.3 [1.0–1.5]
Men (*n* = 15)	0.9 [0.6–1.2]	1.3 [0.7–1.7]
Women (*n* = 15)	1.5 [1.1–1.8]	1.3 [1.0–1.4]

Median [interquartile range]

*TSP*, temporal summation of pain; *PBM*, photobiomodulation

In Table 5, the TSP ratios in the control session are classified as TSP ratios >2 and TSP ratios ≤2. The changes in the TSP ratio by irradiation were classified into a decrease in the TSP ratio of >40% and <40% in the PBM session compared with the TSP ratio in the control session. Three out of the five participants with a TSP ratio >2 in the control session demonstrated a >40% decrease in the TSP ratio in the PBM session. On the other hand, 23 out of the 25 participants with a TSP ratio <2 in the control session demonstrated a <40% decrease in the TSP ratio in the PBM session, and Fisher’s exact test indicated a significant difference (*p* = 0.0219). The frequency plots of individual TSP ratios in the control and PBM sessions showed no differences in men and women.

**Table 5:** TSP ratio in the control session and changes in the TSP ratio by irradiation

	Number of participants who had more than a 40% decrease in the TSP ratio in the PBM session compared with the TSP ratio in the control session	Number of participants who had less than a 40% decrease in the TSP ratio in the PBM session compared with the TSP ratio in the control session
TSP ratio in the control session >2	3	2
TSP ratio in the control session ≤2	2	23

*p* = 0.0219 (Fisher’s exact test)

*TSP*, temporal summation of pain; *PBM*, photobiomodulation

### OA score

**Fig. 3:**
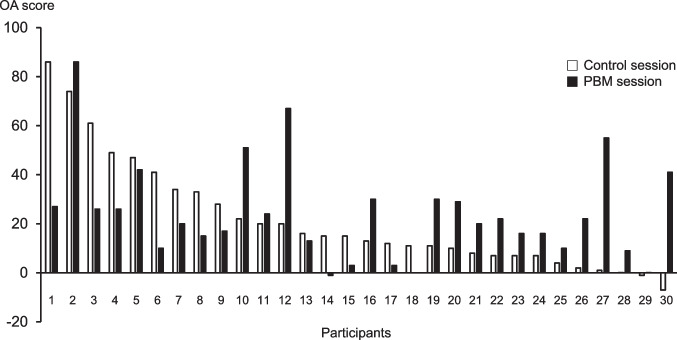
Frequency plot of individual OA scores for 30 participants. Participants are plotted in the order of increasing OA scores in the control session. White square: control session, solid square: PBM session; OA, offset analgesia; PBM, photobiomodulation

The OA scores of all participants, men, and women are presented in Table 6. The paired *t*-test showed no significant differences between the OA scores in the control and PBM sessions in all participants (*p* = 0.5448), men (*p* = 0.8023), and women (*p* = 0.4730). The frequency plots of individual OA scores in the control and PBM sessions in all participants are shown in Fig. 3. Participants with low OA scores in the control session showed improvement in their OA scores in the PBM session. In Table 7, the OA scores in the control session are classified into OA scores >10 and OA scores ≤10, and the changes in OA scores by irradiation were classified into more than twofold and less than twofold increases in OA scores in the PBM session compared with the scores in the control session. Nine out of the 11 participants with OA scores less than 10 in the control session showed more than a twofold increase in their scores in the PBM session compared with their scores in the control session. In contrast, 15 out of the 19 participants with OA scores >10 in the control session showed less than a twofold increase in their scores in the PBM session compared with the scores in the control session, and Fisher’s exact test showed a significant difference (*p* = 0.0021). The frequency plots of individual OA scores in the control and PBM sessions showed no differences in men and women.

**Table 6:** OA scores in the control session and PBM session

	Control session	PBM session
All (*n* = 30)	14.0 [7.0–31.8]	21.0 [10.8–29.8]
Men (*n* = 15)	13.0 [7.5–44.0]	26.0 [15.0–35.5]
Women (*n* = 15)	16.0 [7.0–25.0]	16.0 [11.5–25.0]

Median [interquartile range]

*OA*, offset analgesia; *PBM*, photobiomodulation

**Table 7:** OA scores in the control session and changes in OA scores by irradiation

	Number of participants who had more than a twofold increase in OA scores in the PBM session compared with OA scores in the control session	Number of participants who had less than a twofold increase in OA scores in the PBM session compared with OA scores in the control session
OA score in the control session >10	4	15
OA score in the control session ≤10	9	2

*p* = 0.0021 (Fisher’s exact test)

*OA*, offset analgesia; *PBM*, photobiomodulation

## Discussion

PBM using LPNILI at acupuncture points LI4 and LI10 resulted in a significant increase in the CPM effect in all participants and women, a decreased TSP in participants with higher TSP ratios, and an increased OA in participants with lower OA scores. These findings indicate that PBM via photonic stimulation of acupuncture points enhances endogenous pain modulation in healthy volunteers. In particular, the increased CPM implies that the photonic stimulation in this study strengthened some aspects of endogenous pain modulation evaluated using CPM. In addition, the decrease in TSP in participants with higher TSP ratios and the increase in OA in participants with lower OA scores suggest that the photonic stimulation of acupuncture points enhanced some endogenous pain modulatory mechanisms in participants with weakened endogenous pain modulatory systems, which TSP and OA reflect.

The therapeutic effects of PBM, LAT, and acupuncture differ in individuals [1, 5–8], and individual differences exist in CPM, TSP, and OA [17, 21, 31]. Acupuncture therapy has an analgesic effect; it enhances the descending pain inhibitory mechanisms and alleviates central sensitization [32], which results in the activation of endogenous pain modulation. A recent randomized controlled trial revealed that electro-acupuncture alleviates pain intensity in patients with knee osteoarthritis by improving CPM function [33].

CPM is the term used to describe diffuse noxious inhibitory control (DNIC) in humans [12]. Le Bars et al. were the first to report DNIC in 1979 in animals [34]. DNIC is a phenomenon whereby the activities of convergent neurons in the spinal dorsal horn and trigeminal nucleus are inhibited selectively and powerfully by the application of noxious stimuli in areas that are distant from their excitatory receptive fields [34]. In animal and human studies, serotonergic and noradrenergic neurons are involved in the manifestation of CPM [13–15]. Regarding the gender difference in CPM, some reports indicated attenuated CPM in women compared with that in men [35], whereas others suggested no difference [16]. Tousignant-Laflamme and Marchand evaluated excitatory and inhibitory pain mechanisms during the menstrual cycle in healthy women and demonstrated that women have greater CPM in the ovulatory phase [36]. Moreover, a study on the investigation of the influence of oral contraceptives on CPM in healthy women showed that the decrease in CPM was larger in the no-oral contraceptives group, which indicates that endogenous pain modulation may be less effective in oral contraceptive users [37]. Although hormones, opioids, genotype, attentional components, and other factors are important contributors to the differences observed between men and women [35], gender differences in CPM remain unestablished [38].

PBM is effective for pain relief because it inhibits the action potentials in the dorsal roots [3] and the nociceptive signals in peripheral nerves [4]. Recently, a randomized clinical trial showed that LAT (laser irradiation to ST6, ST7, and LI4) provides pain reduction efficacy similar to that of PBM-associated pain reduction in patients with temporomandibular disorders [39]. Therefore, LAT could be a suitable alternative therapy to PBM [39]. Moreover, gender differences in PBM and LAT outcomes have recently been reported [40, 41]. A recent review evaluating gender differences in responses to PBM therapy, including laser acupuncture, demonstrated that gender-related effects are significant in PBM outcomes, including differences in tendon structural and mechanical outcomes and mitochondrial gene expression [40]. Moreover, a study on gender differences in laser acupuncture outcomes demonstrated that the effects of laser stimulation on the ear were significantly more pronounced in women than in men [41].

A literature review indicated the involvement of endogenous opioid peptides in acupuncture analgesia [42]. LI4 is located between the first and second metacarpal bones. A study that used functional magnetic resonance imaging showed that the distribution of the signally deactivated area evoked by electro-acupuncture on the LI4 point is similar to the known distribution of the anatomical orientation of pain in the brain and closely related to the anatomic structure of the limbic system, which is possibly the cerebral regulating area of the acupuncture analgesic effect [43]. LI10 is located on the forearm, with the arm stretched, three finger’s breadths below Quichi (LI11), located at the elbow. A clinical study revealed that acupuncture at points LI4 and LI10 assists in local anesthesia [44]. Thus, we selected LI4 and LI10 as acupuncture points to induce endogenous pain modulation in the current study. CPM, TSP, and OA are known to be centrally modulated [12–23]. In this study, CPM, TSP, and OA were evaluated on the dominant forearm. If irradiation was performed ipsilaterally, the evaluations of CPM, TSP, and OA might be affected by the irradiation. Therefore, in order to avoid segmental irradiation effects on CPM, TSP, and OA evaluations, irradiation was instead performed on the non-dominant forearm. Concerning the effect of light irradiation, the penetration of red light (660 nm) was unaffected by skin tone but affected by gender; penetration is decreased in men [45]. This study revealed that the photonic stimulation of acupuncture points increased CPM effects, especially in women. This finding could be because of (1) the deeper penetration of photonic stimulation in women than in men due to lower skin thickness in women, (2) the hormonal effects on CPM, or (3) the gender difference in PBM.

TSP is a process of increased pain sensation by repetitive stimulation and is observed in healthy participants [17]. In contrast, previous studies showed that patients with chronic pain had enhanced TSP compared with that of healthy controls [18]. In addition to CPM, TSP has been widely used to diagnose altered pain processing in patients [17]. A clinical study including patients who underwent total knee replacement surgery revealed that the inhibitory CPM and the facilitated TSP are associated with chronic postoperative pain [46]. TSP is considered to reflect central modulation [17]. Central sensitization associated with TSP and changes in inhibitory modulation are likely to interact because opioid agonists and NMDA receptor antagonists modulate TSP in dorsal horn neurons [18–20]. The current study showed that the photonic stimulation of acupuncture points decreased TSP in participants with facilitated TSP before irradiation. This result implies that the photonic stimulation of acupuncture points weakened central sensitization.

The pain inhibition observed in OA is associated with reward systems and descending pain modulatory systems [21]. Moreover, attenuated OA has been reported in patients with chronic pain [21]. The current study showed that the photonic stimulation of acupuncture points enhanced OA in participants with low OA before irradiation. This result suggests that the photonic stimulation of acupuncture points would enhance the reward systems and descending pain modulatory systems.

Few researchers have reported on gender differences in TSP and OA. However, more pain sensitivity in TSP was observed among women than men [47]. Lean mass is a contributing factor to the gender differences in TSP [48], and women show less pain reduction in OA [49]. Overall, the gender difference in pain perception evaluated using CPM, TSP, and OA remains unclear [50]. In the current study, only CPM, but not TSP and OA, was modulated by the photonic stimulation of acupuncture points in all participants and women. A possible reason for this phenomenon could be that the serotonergic and noradrenergic neurons involved in the CPM mechanism might be modulated to some extent by the photonic stimulation of acupuncture points.

Taken together, these findings implied that the photonic stimulation of acupuncture points enhanced endogenous pain modulation evaluated using CPM, TSP, and OA. CPM, TSP, and OA evaluations are potentially useful for discriminating responders from non-responders to PBM and LAT.

The limitation of this study is that randomization for the order of two sessions (control session and PBM session) was not performed because the study was designed such that all the experiments were completed in one day to examine the effect of PBM without introducing inter-day variation. However, the participants and examiner of CPM, TSP, and OA were blinded to whether it was a control session or a PBM session.

## Conclusion

PBM using photonic stimulation of acupuncture points enhanced endogenous pain modulation in healthy volunteers.

## Data Availability

The data that support the findings of this study are available from the corresponding author (Yuka Oono) upon reasonable request.
